# Complete loss of ATM function augments replication catastrophe induced by ATR inhibition and gemcitabine in pancreatic cancer models

**DOI:** 10.1038/s41416-020-1016-2

**Published:** 2020-08-03

**Authors:** Charles R. Dunlop, Yann Wallez, Timothy Isaac Johnson, Sandra Bernaldo de Quirós Fernández, Stephen T. Durant, Elaine B. Cadogan, Alan Lau, Frances M. Richards, Duncan I. Jodrell

**Affiliations:** 1 Cancer Research UK Cambridge Institute, University of Cambridge, Cambridge, UK; 2 Bioscience, Early Oncology R&D, AstraZeneca, Cambridge, UK; 3 Department of Oncology, University of Cambridge, Cambridge, UK

**Keywords:** Checkpoint signalling, Targeted therapies, Chemotherapy, Pancreatic cancer, Predictive markers

## Abstract

**Background:**

Personalised medicine strategies may improve outcomes in pancreatic ductal adenocarcinoma (PDAC), but validation of predictive biomarkers is required. Having developed a clinical trial to assess the ATR inhibitor, AZD6738, in combination with gemcitabine (ATRi/gem), we investigated ATM loss as a predictive biomarker of response to ATRi/gem in PDAC.

**Methods:**

Through kinase inhibition, siRNA depletion and CRISPR knockout of ATM, we assessed how ATM targeting affected the sensitivity of PDAC cells to ATRi/gem. Using flow cytometry, immunofluorescence and immunoblotting, we investigated how ATRi/gem synergise in ATM-proficient and ATM-deficient cells, before assessing the impact of ATM loss on ATRi/gem sensitivity in vivo.

**Results:**

Complete loss of ATM function (through pharmacological inhibition or CRISPR knockout), but not siRNA depletion, sensitised to ATRi/gem. In ATM-deficient cells, ATRi/gem-induced replication catastrophe was augmented, while phospho-Chk2-T68 and phospho-KAP1-S824 persisted via DNA-PK activity. ATRi/gem caused growth delay in ATM-WT xenografts in NSG mice and induced regression in ATM-KO xenografts.

**Conclusions:**

ATM loss augments replication catastrophe-mediated cell death induced by ATRi/gem and may predict clinical responsiveness to this combination. ATM status should be carefully assessed in tumours from patients with PDAC, since distinction between ATM-low and ATM-null could be critical in maximising the success of clinical trials using ATM expression as a predictive biomarker.

## Background

Pancreatic ductal adenocarcinoma (PDAC) is one of the few major cancers where overall 5-year survival remains <10%.^1^ While surgical resection can be curative, the late emergence of symptoms means that most patients with PDAC have metastatic disease at presentation and are ineligible for surgery.^2^ In the advanced disease setting, the combination chemotherapy regimen FOLFIRINOX (5-FU/leucovorin/irinotecan/oxaliplatin) has been shown to improve survival, but the median overall survival for patients with stage IV disease remains <12 months.^3^ This highlights the need for better therapeutic approaches. It is hoped that the rational selection of patients, through precision medicine approaches, will improve outcomes. However, for personalised treatment strategies to succeed, careful validation of potential predictive biomarkers of response is essential.

Recently, we demonstrated that the ataxia telangiectasia and Rad3-related protein (ATR) kinase inhibitor, AZD6738, synergised with gemcitabine in PDAC models.^4^ A key regulator of the DNA damage response (DDR), ATR is a serine/threonine kinase that coordinates the replication stress (RS) response. Upon stalling of a replication fork, ATR acts locally to stabilise the fork and in addition propagates a global signal throughout the nucleus by phosphorylating several targets, most notably checkpoint kinase 1 (Chk1).^5,6^ When active, Chk1 inhibits dormant origin firing to limit single-stranded DNA (ssDNA) exposure and thus prevents nuclear exhaustion of RPA.^7^ Chk1 induces cell cycle arrest by phosphorylating and inhibiting CDC25 phosphatases.^8,9^ This activation of the *intra-S checkpoint* allows time for attempted restart of any stalled forks and completion of DNA synthesis in the RS-affected regions. The antimetabolite gemcitabine has been used extensively in the treatment of patients with PDAC, either as a single agent or in combination with nab-paclitaxel. As a nucleoside analogue, gemcitabine impairs the replication machinery through multiple mechanisms and induces fork stalling,^10^ which activates the ATR/Chk1 axis. We demonstrated previously that AZD6738 prevents gemcitabine-induced intra-S checkpoint activation in PDAC cells, leading to an induction of double-strand break (DSB) markers.^4^ The combination of AZD6738 and gemcitabine (ATRi/gem) inhibited growth in a panel of PDAC cell lines in vitro, while in vivo the combination induced tumour regression. As a direct result of those studies, a phase I clinical trial has been launched to assess the safety, tolerability and preliminary antitumour activity of ATRi/gem in participants with advanced solid tumours, followed by an expansion phase in patients with advanced PDAC (ATRiUM; NCT03669601).

Being a novel combination, there are no established predictive biomarkers of response for ATRi/gem. Knowing that ATRi/gem induces DSBs, we hypothesised that PDAC cells deficient in DDR pathways would be hypersensitive to the combination. We focussed our attention on ataxia telangiectasia mutated (ATM), a DDR kinase that acts as a master regulator of cellular responses to DSBs^11,12^ which is mutated or lost in a proportion of tumours of many cancer types, including in PDAC where it is a known susceptibility gene.^13^ A recent comprehensive literature review of ATM deficiency in PDAC captured 5234 pancreatic cancer patients and estimated that the prevalence of germline or somatic ATM mutations in PDAC was 6.4%, though they uncovered a large range of 1–34%.^14^ In addition, tissue microarray data sets indicate that low ATM protein expression in PDAC occurs at around 12–17%,^15,16^ though there is no consensus for defining “ATM-low”.

The loss of ATM in a proportion of PDAC samples, plus the central role it plays in DSB repair, make ATM a primary candidate for a potential predictive biomarker of response for ATRi/gem therapy. Loss or mutations in ATM have previously been linked, in some cancer models, to greater sensitivity to ATRi monotherapy. For example, primary CLL cells with ATM defects showed enhanced sensitivity to AZD6738 compared to wild-type (WT) or normal cells^17^, and when ATM was knocked down with small interfering RNA (siRNA) in the gastric adenocarcinoma line, SNU-484, AZD6738 IC_50_ was reduced ≥2-fold.^18^ In contrast, when Vendetti et al. used short hairpin RNAs to deplete ATM in two lung cancer lines, the shATM cells appeared more resistant to AZD6738 monotherapy than control—only when AZD6738 was combined with cisplatin did shATM sensitise.^19^ In pancreatic cancer models, Ayars et al. reported that MIA PaCa-2 cells treated with shATM were no more sensitive to the ATR inhibitor VX-970/VE-822 analogue, VE-821, than control cells.^20^ However, work by Perkhofer et al. using mouse models of ATM-deficient PDAC suggested that ATM loss did confer sensitivity to VE-822.^21^

This lack of consistency in the literature between cancer model, ATR inhibitor used and the experimental method of ATM depletion employed led us to undertake an assessment of how ATM status affects ATRi sensitivity in PDAC cells, specifically using AZD6738 with and without gemcitabine, in alignment with the ATRiUM trial. By targeting ATM through multiple methods, we reveal that complete loss of ATM function—through kinase inhibition or through CRISPR knockout (KO), but not ATM depletion by RNA interference—sensitises to ATRi and ATRi/gem in PDAC models. We gained further insight into the mechanisms by which AZD6738 and gemcitabine synergise to induce cell death, finding evidence for replication catastrophe (RC) that was significantly augmented in ATM-deficient cells. Our results suggest that ATM status should be carefully assessed in tumours from patients with PDAC and that the distinction between ATM-low and ATM-null could be crucial in maximising the success of trials using ATM expression as a predictive biomarker.

## Methods

### Cell culture and chemicals

Human cell lines were purchased from ATCC and cultured in Dulbecco’s modified Eagle’s medium (DMEM; ThermoFisher#41966029) (MIA PaCa-2, PANC-1 and HPAF-II) or RPMI-1640 (ThermoFisher#21875034) (AsPC-1) plus 10% foetal bovine serum (FBS; ThermoFisher#10270106). Murine cell lines K8484 and DT8082 were previously established from KPC mice of 129/SvJae/C57Bl/6 background in the laboratory of David Tuveson at the CRUK Cambridge Institute^22^ and were grown in DMEM with 5% FBS. Lines were subjected to regular STR fingerprinting and mycoplasma tests, performed by the CRUK-CI Cell Services core facility. AZD6738, AZD0156 and AZD7648 were provided by AstraZeneca. These, plus gemcitabine hydrochloride (Tocris#3259), were dissolved in dimethyl sulfoxide (DMSO) for in vitro experiments, kept at −20 °C and used within 3 months.

### siRNA transfection

ON-TARGETplus-SMARTpool siRNAs targeting ATM were purchased from Dharmacon (#L-003201-00-0005), as well as a Non-Targeting-Control-Pool (#D-001810-10-05). Reverse transfection was achieved using Lipofectamine RNAiMax reagent (Invitrogen#13778/150), as per the manufacturer’s instructions. Briefly, lipofectamine–siRNA mixture was incubated in a 100-mm dish, before cells were seeded on top (final siRNA concentration = 25 nM). Forty-eight hours later, cells were split and seeded for drug sensitivity assays or immunoblotting.

### CRISPR/Cas9 gene editing

A previously validated all-in-one gRNA-Cas9-GFP plasmid (pAiO-WT-ATM), the human ATM-specific gRNA sequence, GTTGGTTACATACTTGGACT, cloned into the BbsI site, was kindly provided by Professor Stephen Jackson of the Wellcome Trust/CRUK Gurdon Institute, University of Cambridge, UK.^23^ MIA PaCa-2 forward transfection was achieved using Lipofectamine 3000 (Invitrogen#L3000015) as per the manufacturer’s instructions (6-well plate). Forty-eight hours post-transfection, cells were split and single-cell sorted (BD FACS Aria), specifically for the top 3% of green fluorescent protein (GFP)-positive cells, to enrich for those positively transfected. Clones were bulked up, before half of each sample was taken for genomic DNA extraction (QIAamp DNA Micro Kit #56304). From genomic DNA, the region around the sgATM-Cas9 target site was amplified by PCR and sent for Sanger nucleotide sequencing (Eurofins LightRun). Sequencing chromatograms were deconvoluted using the Synthego ICE web tool (ice.synthego.com). Absence of ATM protein was confirmed by immunoblotting.

### Immunoblotting

Following media removal and a phosphate-buffered saline (PBS) wash, cell lysis was performed on 60-mm dishes using 50 mM Tris HCl plus 2% sodium dodecyl sulfate (SDS), with phosphatase and protease inhibitors (Roche#04906837001, Roche#04693159001). Cells were scraped and boiled at 95 °C for 5 min, before NanoDrop™ 8000 (A280) protein quantification. Proteins were resolved using the SDS-polyacrylamide gel electrophoresis gel system (Life Technologies), detected using IRDye secondary antibodies (LI-COR) and visualised on the Odyssey CLx imaging system (LI-COR). Primary antibodies used were obtained from Abcam (ab) or Cell Signaling Technology (CST), unless otherwise stated: ATM (ab78), p-ATM-Ser1981 (ab81292), DNA-PKcs (ab44815), P-DNA-PKcs-Ser2056 (ab18192), KAP1 (ab22553), P-KAP1-Ser824 (ab133440), RPA32 (ab2175), RAD50 (ab89), p-Chk2-Thr68 (ab3501), Chk2 (CST3440), p-Rad50-Ser635 (CST14223S), Chk1 (CST2360), p-Chk1-Ser345 (CST2348), H2AX (CST7631), β-Actin (CST4970), β-Tubulin (CST2146), Lamin B1 (CST12586), Vinculin (CST13901), ATR (Santa Cruz Biotechnology#SC-1887), p-ATR-Thr1989 (Genetex#GTX128145), γH2AX-Ser139 (Millipore#05-636), p-RPA32-Ser4/8 (Bethyl#A300-245A).

### Drug sensitivity assays

Sulforhodamine B (SRB) assays, clonogenic assays and IncuCyte time-lapse imaging experiments were performed as we have described previously.^4^

### Flow cytometry

Cells were plated in Corning 60-mm dishes 24 h prior to drug treatment. After treatment, cells were trypsinised, fixed with ice-cold 70% EtOH overnight at −20 °C, washed, then resuspended in 0.5 mL of blocking solution (BS) (PBS, 2% BSA, 0.1% Tween-20, 0.1% Triton X100) for 1 h. Finally, cells were treated with 0.5 mL FxCycle Violet Stain (Invitrogen #F10347), in BS at 1:1000 dilution, and run on the BD Biosciences LSRFortessa™ flow cytometer. The resulting data were analysed using the FlowJo® V10 software.

### Quantitative image-based cytometry

Cells were seeded in Corning black flat-bottom 96-well plates 24 h before drug treatment. After treatment, cells were fixed with 4% paraformaldehyde for 10 min, permeabilised with 0.1% Triton X-100, 0.1% Tween-20, and 1×PBS (PBSTT) for 10 min at room temperature and blocked for 30 min with BS (same as flow). Primary antibody in BS was added for 1 h at room temperature (mouse anti-γH2AX S139, Millipore #05-636, 1:2000 and rabbit anti-phospho-RPA32 S4/8, Bethyl#A300-245A, 1:1000). After washing, secondary antibody (Alexa Fluor 488 goat anti-rabbit, Invitrogen#A11034, 1:500 and Alexa Fluor 568 goat anti-mouse, Invitrogen#A11019, 1:500) plus Hoechst 33342 (Invitrogen#H3570, 1:10,000) in BS was added for 1 h at room temperature. After washing, images were acquired using the Operetta CLS High-Content Analysis System (Perkin Elmer), and analysis was performed using the Harmony 4.5 software.

### Fractionation

Fractionation, to derive cytoplasmic and nuclear fractions, was performed as described by Warren and Eastman.^24^

### Animal experiments

All mouse experiments were carried out in the CRUK-CI BRU, in accordance with the UK Animals (Scientific Procedures) Act 1986, with approval from the CRUK-CI Animal Welfare and Ethical Review Body. Subcutaneous xenografts of MIA PaCa-2 cells were formed by implanting 5 × 10^6^ cells (in 0.2 mL 1:1 Matrigel: PBS) in the flank of 7–9-week-old female NSG mice (Charles River, 5 mice per cage). Despite some limitations (lack of intratumoural heterogeneity, absence of immune responses, reduced ability to recapitulate the tumour microenvironment), subcutaneous xenografts can offer some advantages over orthotopic models, as previously reported,^25^ including ease of use, reproducibility and earlier tumour detection. Mice with established tumours (average 242 mm^3^) were randomised into treatment groups using the “spiral” randomisation method (see Supplementary Material). AZD6738 (AstraZeneca) was dissolved to 5 mg/mL in 10% DMSO, 40% propylene glycol and 50% de-ionised sterile water and given at 50 mg/kg by oral gavage. Gemcitabine (LKT Laboratories, from Cambridge Bioscience) was dissolved in sterile saline (Vetivex) to 10 mg/mL and given at 50 mg/kg intraperitoneally (IP). Tumours were measured twice a week using callipers. Tumour growth inhibition (TGI) = [MeanVolCtrl − MeanVolTreated/MeanVolCtrl] × 100. At endpoint, blood was collected by cardiac puncture (under terminal anaesthesia using isoflurane in accordance with NC3Rs guidelines) and run on the Mythic 18 haematology analyser as per instructions (Woodley Equipment Co. Ltd). Mice were killed by cervical dislocation, confirmed by cutting the femoral artery.

### Immunohistochemistry (IHC)

Formalin-fixed, paraffin-embedded sections were immunostained after heat-induced epitope retrieval by sodium citrate at 100 °C for 10–20 min, using the Bond Polymer Refine Detection Kit on the automated Bond system according to the manufacturer’s instructions (Leica Biosystems). Slides were mounted using Leica CV5030 Coverslipper Workstation and scanned using a ScanScopeXT (Aperio Technologies). Quantification was performed using the Halo software (Indica Labs). γH2AX S139 primary antibodies were CST9718 on human xenograft tissues and ab195190 on mouse intestine.

## Results

### Pharmacological inhibition of ATM sensitises to ATR inhibition in PDAC cell lines

**Fig. 1:**
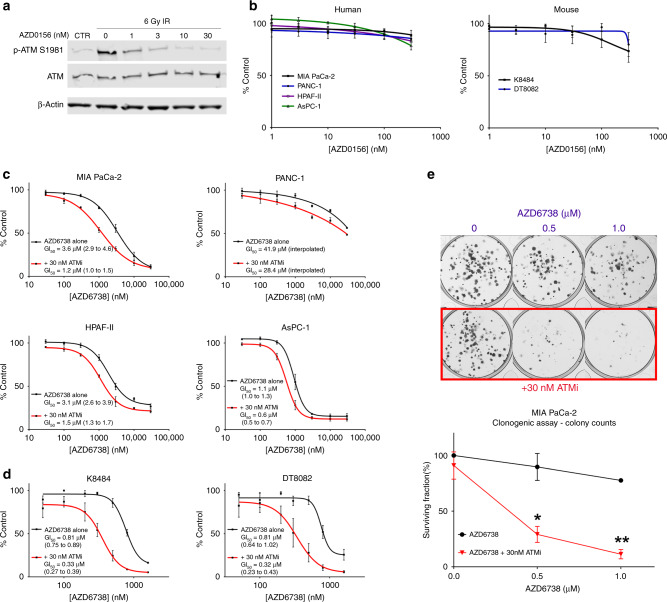
Pharmacological inhibition of ATM sensitises to ATR inhibition in PDAC cell lines. **a** MIA PaCa-2 were incubated with AZD0156 for 1 h before exposure to 6 Gy of γ-irradiation (IR). Thirty minutes post-IR, cells were harvested for immunoblot analysis. **b** Human and mouse cell lines were exposed to AZD0156 for 72 h to generate dose–response curves using the SRB assay. Each point represents the mean ± SEM of three independent experiments. **c**, **d** AZD6738 dose–response curve with and without 30 nM AZD0156, mean ± SEM, *n* = 3, with GI_50_ plus 95% confidence intervals shown. Human (**c**) and mouse (**d**) lines were treated for 72 h. **e** Clonogenic survival of MIA PaCa-2 cells plated at low density and exposed to the indicated drug combinations for 24 h before washout. Cells were left to grow for 7 days after washout. Each point represents the mean ± SEM of three independent experiments. The difference between *AZD6738* and *AZD6738* + 30 nM ATMi was assessed using multiple *t* tests. *p ≤ 0.05, **p ≤ 0.01.

To assess the degree of sensitisation to ATR inhibition associated with loss of ATM function in PDAC cells, we used the selective and potent ATM kinase inhibitor, AZD0156,^26,27^ in combination with ATR inhibitor AZD6738.^28^ First, to confirm target engagement, we assessed the ability of AZD0156 to prevent ATM activation in PDAC cells. Irradiation (IR) 6 Gy with analysis of P-ATM-S1981 at 0.5–1 h has previously been used by other groups to study ATM activation in human cancer cells.^27,29–31^ In MIA PaCa-2, low nanomolar concentrations of AZD0156 (≥10 nM) successfully abrogated IR-induced ATM auto-phosphorylation (Fig. 1a). In the absence of extrinsic damage, AZD0156 exposure had minimal effect on human or mouse PDAC cell growth when used at concentrations ≤100 nM (Fig. 1b), at which off-target activity is minimised. We next evaluated the degree of growth inhibition induced by AZD6738 (ATRi) across a range of AZD0156 (ATMi) concentrations. ATMi 30 nM sensitised all six of the human (Fig. 1c) and mouse (Fig. 1d) PDAC lines tested to ATR inhibition. Calculation of Bliss and Loewe synergy scores using the Combenefit software^32^ showed that the ATRi/ATMi combination synergistically inhibited growth in all six of these lines, albeit modestly in the ATRi-resistant PANC-1 (GI_50_ > 30 µM; Fig. S1). To assess the long-term proliferation capacity of cells exposed to a 24-h pulse of AZD6738, we then performed clonogenic assays. In this assay, ATMi strikingly sensitised MIA PaCa-2 to ATRi (Fig. 1e).

### ATM protein depletion by siRNA knockdown does not sensitise PDAC cells to ATR inhibition

**Fig. 2:**
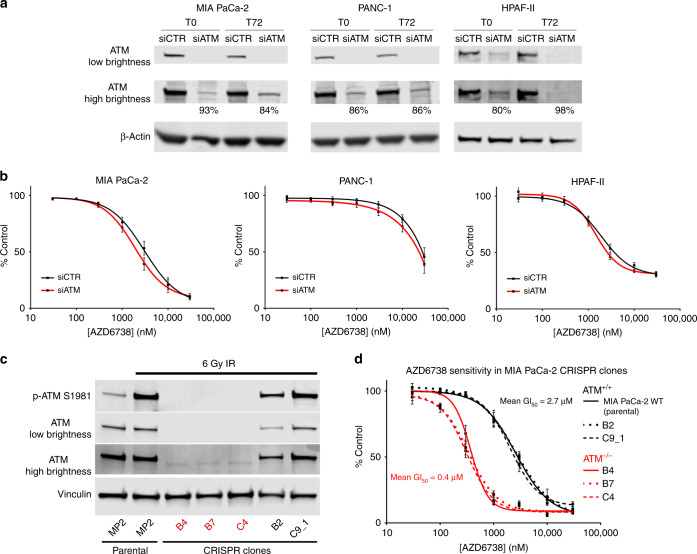
Deletion of ATM using CRISPR/Cas9, but not protein depletion by siRNA knockdown, sensitises PDAC cells to ATR inhibition. **a** ATM protein expression in human cell lines at the start (T0) of the drug sensitivity assay (48 h post-transfection) and at the 72-h assay endpoint (120 h post-transfection). Two different “exposures” of the ATM blot are shown (by adjusting brightness of the IRDye image in LiCor Image Studio). Percentage knockdown values vs siCTR are displayed, derived using the LiCor Image Studio quantification software. **b** AZD6738 dose–response curves of human lines, having been transfected with either a non-targeting siRNA control pool (siCTR) or with an ATM-targeting siRNA pool (siATM). Assay duration was 72 h. Each point represents the mean ± SEM of three independent experiments. **c** Thirty minutes post 6 Gy of γ-irradiation (IR), MIA PaCa-2 single-cell clones from a CRISPR/Cas9 ATM knockout pool were harvested for immunoblot analysis to determine the ATM status. Clones B4, B7 and C4 were confirmed ATM-null. **d** AZD6738 dose–response curves of MIA PaCa-2 CRISPR clones. Assay duration was 72 h. Each point represents the mean ± SEM of three independent experiments.

Having identified that pharmacological inhibition of ATM in PDAC cell lines can sensitise to ATRi, we next evaluated the potential for siRNA knockdown of ATM to confer ATRi sensitivity in three human cell lines—MIA PaCa-2, HPAF-II and PANC-1. Despite achieving durable ATM knockdown (88% mean knockdown efficiency across the 3 lines, SD ±7%) (Fig. 2a), this did not significantly sensitise any of the 3 cell lines to 72-h exposure to AZD6738 (Fig. 2b). Due to this disparity between kinase inactivation (ATMi) and protein depletion (siATM), we hypothesised that reduced expression of ATM may be insufficient to sensitise cells to AZD6738.

### Deletion of ATM using CRISPR/Cas9 does significantly sensitise PDAC cells to ATR inhibition

Having observed a sensitisation effect with the ATMi, but not with siATM, we next used CRISPR/Cas9 technology to generate ATM KO MIA PaCa-2 single-cell clones. By Sanger sequencing (Fig. S2A) and immunoblotting, we identified three clones that did not express detectable ATM and showed no phospho-ATM S1981 upon IR (Fig. 2c). These three ATM-null clones were sevenfold more sensitive to AZD6738 than WT MIA PaCa-2 cells (mean ATM-null cells GI_50_ = 0.37 µM; mean ATM-positive cells GI_50_ = 2.73 µM; Figs. 2d and S2B). As sensitisation occurred upon ATM KO, but not knockdown with siRNA, this suggests that complete loss of ATM function, not just depletion, is necessary to sensitise PDAC cells to ATRi monotherapy.

### ATM loss of function sensitises to the combination of AZD6738 and gemcitabine

**Fig. 3:**
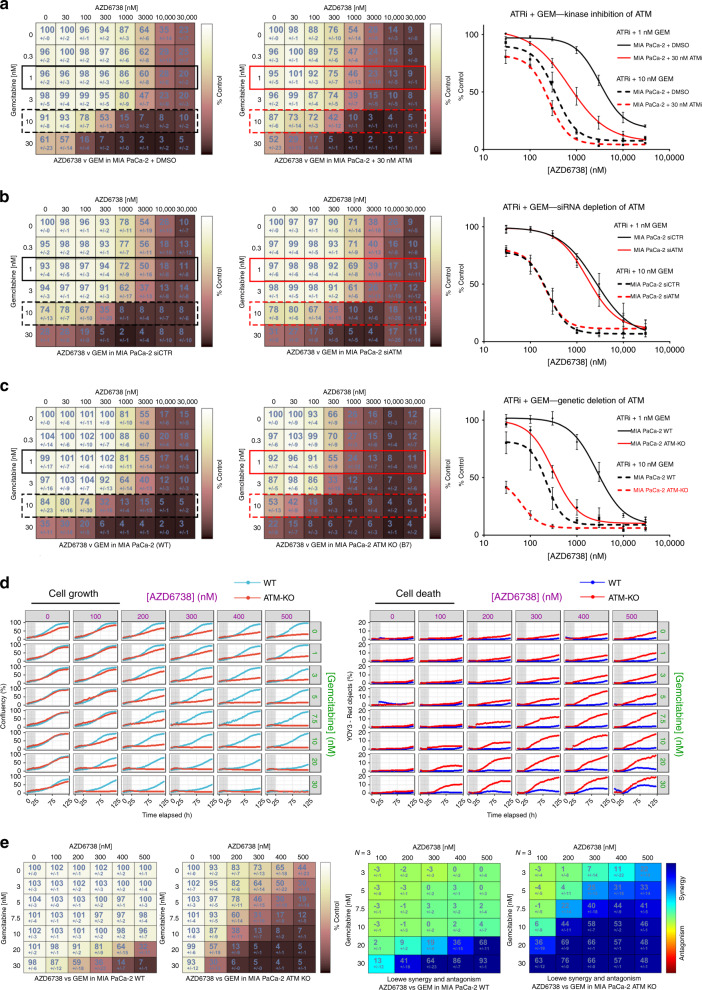
ATM loss of function sensitises to the combination of AZD6738 and gemcitabine. **a** The effect of ATM kinase inhibition by AZD0156 on ATRi (AZD6738) and gemcitabine sensitivity in MIA PaCa-2. **b** The effect of ATM depletion by siRNA on AZD6738 and gemcitabine sensitivity in MIA PaCa-2. **c** The effect of ATM deletion by CRISPR/Cas9 on AZD6738 and gemcitabine sensitivity in MIA PaCa-2. In all three panels (**a**–**c**), MIA PaCa-2 in the conditions listed were treated with increasing concentrations of AZD6738 and gemcitabine for 72 h. Left = matrices display growth as percentage of solvent control (as assessed by SRB assay), mean ± SD, *n* = 3. Right = AZD6738 dose–response curves in the presence of 1 or 10 nM gemcitabine, mean ± SEM, *n* = 3. **d** MIA PaCa-2 WT and MIA PaCa-2 ATM-KO (clone B7) cells in medium containing the YOYO-3 iodide cell-impermeant dye were treated with AZD6738 and gemcitabine in a 6 × 8 concentration grid for 24 h (grey bar denotes period of drug treatment). The drugs were washed out and replaced with fresh YOYO-3-containing medium. Three fields per sample were imaged by IncuCyte time-lapse microscopy every 3 h for 4 days post-washout. Phase Object Confluence was quantified as the percentage of the image area occupied by cells (left). Cell death accumulation (right) was quantified by measuring Red Object Confluence, and expressed as a percentage of the total Object Confluence. Each curve is a representative of three independent experiments. **e** The same cells from the IncuCyte experiment in **d** were fixed in TCA 4 days post-washout and the relative growth was assessed by SRB assay. Left = growth as percentage of solvent control, mean ± SD, *n* = 3 independent experiments. Right = Combenefit synergy score (Loewe).

We next tested whether reduced ATM function could sensitise to the combination of AZD6738 and gemcitabine (ATRi/gem). Kinase inhibition of ATM conferred sensitivity to 72-h ATRi/gem exposure in MIA PaCa-2 (Fig. 3a) but siRNA depletion did not (Fig. 3b). Meanwhile, genetic deletion of ATM caused a 2.6-fold shift in the mean gemcitabine GI_50_ (Fig. S2B, C) and made MIA PaCa-2 cells hypersensitive to ATRi/gem (Fig. 3c). This was particularly evident in assays where the ATRi/gem was pulsed for 24 h with observation of subsequent cell growth by time-lapse imaging. A 24-h pulse of 500 nM AZD6738 and 10 nM gemcitabine had no effect on the growth ability of the WT line (growth as percentage of solvent control = 96% ± 7) but maintained durable growth inhibition for at least 4 days in the ATM-KO cells (growth as percentage of solvent control = 7% ± 1) (Fig. 3d, e), with a parallel increase in cell death, quantified by YoYo-3 staining (Fig. 3d). The impressive sensitivity of the isogenic ATM-KO cells to the combination of AZD6738 and gemcitabine provides evidence that complete ATM loss could be a predictive biomarker of response to ATRi/gem in patients with PDAC.

### ATRi/gem-induced DDR activation persists in the absence of ATM function due to DNA-PK activity

**Fig. 4:**
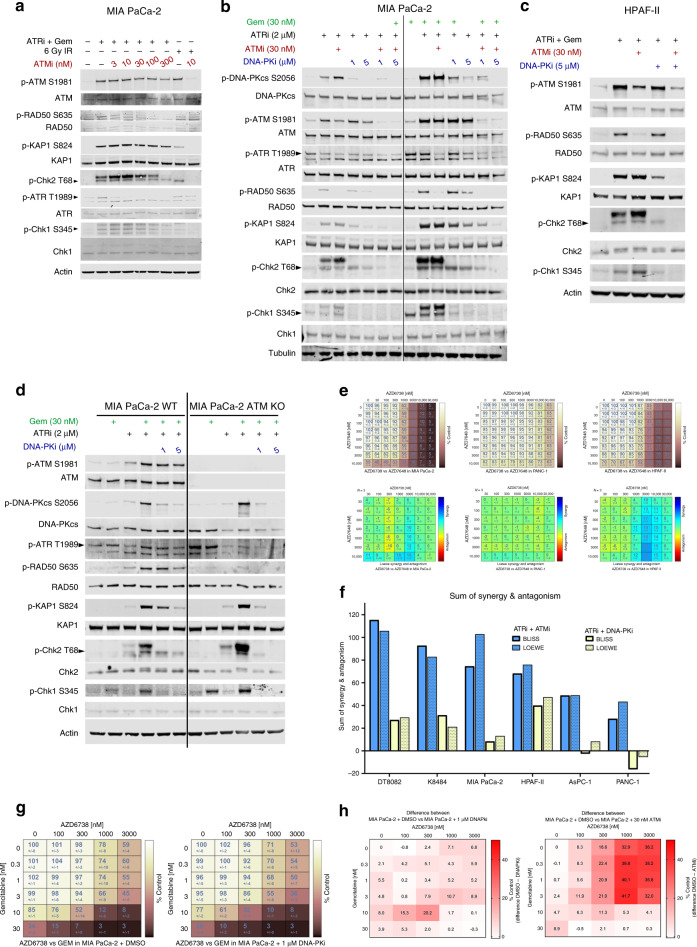
ATRi/gem-induced DDR activation persists in the absence of ATM function due to DNA-PK activity. **a** Immunoblot analysis of MIA PaCa-2 cells. Cells that received 6 Gy of γ-irradiation (IR) were harvested 30 min post-IR. All other samples were harvested after 24 h of drug exposure. ATRi + GEM denotes 2 µM AZD6738 and 30 nM gemcitabine. ATMi is AZD0156 and was either applied concurrently with ATRi/gem or administered 1 h prior to IR in the case of the two IR samples. **b** Immunoblot analysis of MIA PaCa-2 cells. Cells were harvested after 24 h of drug exposure. ATRi is AZD6738, ATMi is AZD0156, DNA-PKi is AZD7648. **c** Immunoblot analysis of HPAF-II cells. Cells were harvested after 24 h of drug exposure. **d** Immunoblot analysis of MIA PaCa-2 WT and MIA PaCa-2 ATM-KO cells (clone B7). Cells were harvested after 24 h of drug exposure. **e** Cells were treated with increasing concentrations of ATRi (AZD6738) and DNA-PKi (AZD7648) for 72 h, and growth was assessed by SRB assay. Top = growth as percentage of solvent control. Bottom = Combenefit synergy score (Loewe). Data, mean ± SD, *n* = 3. For matrices of all cell lines, see Fig. S3A. **f** Synergy comparison of the AZD6738 and AZD0156 combination (ATRi + ATMi, see Fig. S1) vs the AZD6738 and AZD7648 combination (ATRi + DNAPKi, Fig. S3A). Cells were treated with increasing concentrations for 72 h and growth as percentage of solvent control was assessed by SRB assay. Combenefit software was used to calculate the sum of synergy and antagonism across the dose ranges tested. **g** The effect of DNA-PK inhibition on AZD6783 and gemcitabine sensitivity in MIA PaCa-2. Values denote growth as percentage of solvent control (as assessed by SRB assay), mean ± SD, *n* = 3. **h** Visualisation of how much more effect ATM inhibition has compared to DNA-PK inhibition on the growth of ATRi/gem-treated MIAPaCa-2. Left = the %Control values of the MIA PaCa-2 + 1 µM DNA-PKi matrix from Fig. 4g were subtracted from those of the MIA PaCa-2 + DMSO matrix from Fig. 4g. Right = the %Control values of the MIA PaCa-2 + 30 nM ATMi matrix from Fig. 3a were subtracted from those of the MIA PaCa-2 + DMSO matrix from Fig. 3a.

Having found that targeting ATM, by either pharmacological inhibition or by genetic deletion, can sensitise PDAC cells to the combination of AZD6738 and gemcitabine, we next investigated the DDR signalling pathways activated by ATRi/gem, in the presence or absence of ATM function. Twenty-four-hour exposure of MIA PaCa-2 cells to 2000 nM ATRi and 30 nM gemcitabine (concentrations known to be synergistic in this line^4^) induced phosphorylation of ATM and its downstream targets RAD50, KAP1 and Chk2 (Fig. 4a). Perhaps paradoxically (since they are reported to be ATM targets), phospho-ATM S1981, phospho-KAP1 S824 and phospho-Chk2 T68 all persisted even in the presence of ATMi (AZD0156) (Fig. 4a). This persistence was specific to ATRi/gem, since IR-induced activation of these markers was prevented by 10 nM ATMi (Figs. 1a and 4a). This is similar to our previous findings, when we found that the ATRi/gem combination induced P-Chk2 and P-KAP1 to a similar extent in siATM cells as in siCTRL cells.^4^ Here, in the ATRi/gem treated samples, we did observe ATMi dose-dependent abrogation of phospho-RAD50 S635 (Fig. 4a), a biomarker of ATM activity,^33,34^ suggesting that the ATMi was acting on target. Therefore, the persistence of phospho-Chk2 T68 and others could be due to the activity of another kinase, the most likely candidate being DNA-PK. Indeed, in both MIA PaCa-2 (Fig. 4b) and HPAF-II (Fig. 4c), the phospho-KAP1 and phospho-Chk2 induced by ATRi or ATRi/gem treatment was abrogated by the selective DNA-PK inhibitor, AZD7648.^35^ Along with phospho-ATM, these phosphorylations were only fully prevented upon combined ATMi and DNA-PKi. We also probed for ATR and its downstream partner Chk1. Gemcitabine-induced phospho-ATR T1989 persisted in the presence of ATRi due to ATM-dependent phosphorylation (Fig. 4b). Meanwhile, phospho-Chk1 S345 was only abrogated upon DNA-PK inhibition (Fig. 4b, c). Thus we revealed that much of the downstream DDR activation induced by ATRi or ATRi/gem was not prevented by ATMi (besides phospho-RAD50 and phospho-ATR) because of phosphorylation by DNA-PK.

We next interrogated DDR pathway activation in ATM-KO MIA PaCa-2 cells. Once again, ATRi/gem treatment upregulated phospho-Chk2, phospho-KAP1 and phospho-Chk1 in both the presence and absence of ATM function (Fig. 4d). These phosphorylations were DNA-PK driven, as evidenced by their abrogation by AZD7648 (Fig. 4d). The low baseline levels of phospho-RAD50 S635 in untreated ATM-KO MIA PaCa-2 cells appeared to be ATR dependent, consistent with recent findings that in ATM-deficient models ATR can phosphorylate RAD50.^34^ Unlike with Chk2 and KAP1, the ability for the ATM-KO cells to upregulate phospho-RAD50 S635 upon ATRi/gem was significantly impaired in comparison to ATM-WT. The modest phospho-RAD50 S635 that was induced by ATRi/gem was DNA-PK dependent (Fig. 4d). Meanwhile, phosphorylation of ATR T1989 appeared to be ATR and ATM dependent but not DNA-PK dependent (Fig. 4d).

Despite the contribution of DNA-PK to many of the DDR phosphorylations in ATRi-treated cells, Bliss and Loewe cytotoxicity synergy scores showed consistently less synergy for the AZD6738 and AZD7648 (DNA-PKi) combination than for AZD6738 and AZD0156 (ATMi) across human and mouse PDAC cell lines (Figs. 4e, f and S3A). Furthermore, in triple combination SRB experiments, the addition of DNA-PKi to ATRi/gem led to only moderate sensitisation compared to ATMi (Figs. 4g, h and S3D). This suggests that ATM function is more critical to cell survival during ATRi or ATRi/gem exposure than DNA-PK activity. It also implies that the Chk1/Chk2 phosphorylations that arise through DNA-PK activity do not play a major protective role in ATRi/gem-exposed PDAC cells.

### ATRi/gem-induced RC is augmented in ATM-null PDAC cells

**Fig. 5:**
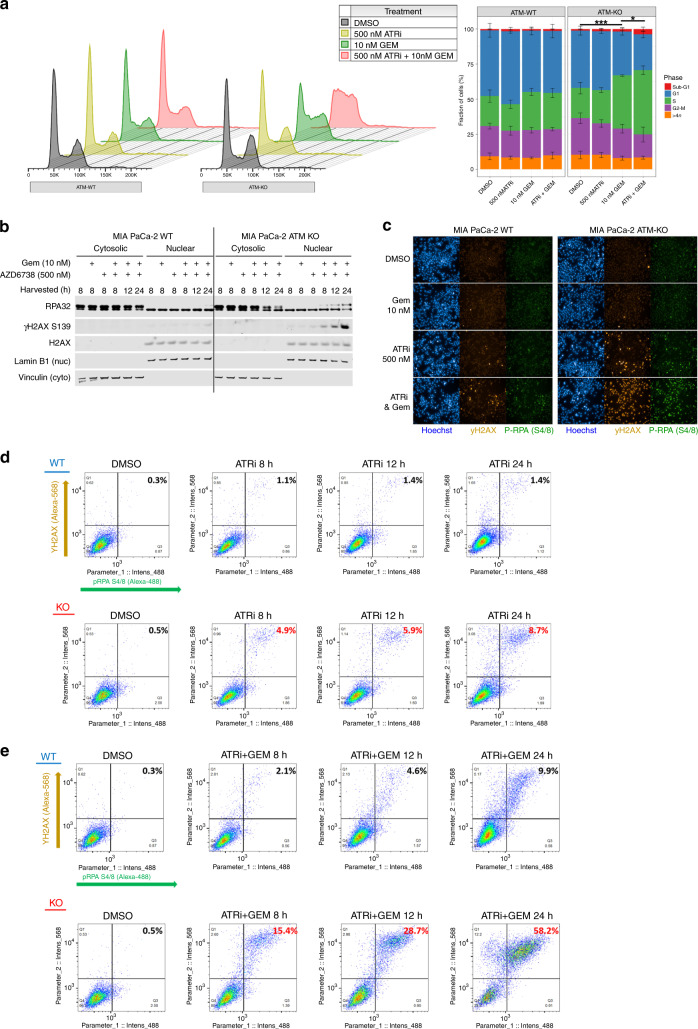
ATRi/gem-induced replication catastrophe is augmented in ATM-null PDAC cells. **a** DNA content of MIA PaCa-2 WT and MIA PaCa-2 ATM-KO (clone B7) cells following 24-h exposure of the indicated drugs. Left = representative DNA content histograms, *x* = FxCycle Violet fluorescence (DNA content), *y* = cell count. Right = cell cycle distribution. Each bar represents the mean ± SD of three independent experiments. A one-way ANOVA analysis, comparing the percentage of S-phase fractions, was performed with Tukey’s multiple comparisons tests, *p ≤ 0.05, ***p ≤ 0.001. **b** Immunoblot analysis of MIA PaCa-2 WT and MIA PaCa-2 ATM-KO cells treated as indicated, separated by cytosolic and nuclear fractions. **c** Representative images showing the pan-nuclear emergence of γH2AX S139 and phospho-RPA32 S4/8 upon 24-h exposure to 500 nM AZD6738 and 10 nM gemcitabine. The images shown were acquired using a ×10 objective lens. Images acquired using a ×20 objective lens are displayed in Fig. S4B. **d**, **e** Quantitative image-based cytometry to determine the proportion of cells pan-nuclear for both γH2AX S139 and phospho-RPA32 S4/8, at the time points indicated. ATRi = 500 nM AZD6738. GEM = 10 nM gemcitabine. Images were acquired using the Operetta CLS High-Content Analysis System (×10 objective) and analysis was performed using the Harmony 4.5 software. The resulting data were imported into FlowJo as CSV files to generate the pseudo-colour plots shown. Percentages of double-positive cells are shown.

We next investigated the effect of AZD6738 and gemcitabine treatment on the cell cycle profile of ATM-proficient and ATM-deficient PDAC cells. Treatment with 500 nM AZD6738 and 10 nM gemcitabine for 24 h had little-to-no effect on the cell cycle profile of WT MIA PaCa-2 cells. Conversely, 10 nM gemcitabine induced significant intra-S accumulation in the ATM-KO MIA PaCa-2, along with a reduction in the G2-M proportion (Fig. 5a). This was augmented by the addition of 500 nM AZD6738, which also increased the sub-G1 fraction, indicating induction of cell death. When the higher concentration of 2000 nM AZD6738 was used, we observed modest intra-S accumulation with ATRi monotherapy, which was specific to the ATM-null cells, while addition of 30 nM gemcitabine could induce S-phase arrest in both the ATM-WT and ATM-KO cells (Fig. S4A).

Through immunoblotting, we had found that ATRi/gem treatment induced DNA-PK-driven activation of the checkpoint proteins, Chk1 and Chk2. However, co-treating with the DNA-PKi AZD7648 did not alter the cell cycle profiles in any of the ATRi/gem conditions (Fig. S4A), indicating that the intra-S-phase arrests we observed were independent of checkpoint activation. An alternative reason for the intra-S accumulation, independent of a checkpoint, could be induction of RC. This is defined as the widespread breakage of multiple replication forks, resulting from a global exhaustion of RPA and subsequent degradation of unprotected ssDNA at stalled forks.^7,24,36^ We found that 500 nM AZD6738 and 10 nM gemcitabine caused RPA32 to deplete from the cytosol and accumulate in the nucleus over time, with a parallel increase in nuclear γH2AX S139, in the ATM-KO MIA PaCa-2 (Fig. 5b). Next, using quantitative image-based cytometry, we assessed the degree of RC induced by ATRi and ATRi/gem in ATM-WT vs ATM-KO, by quantifying the emergence of cells with pan-nuclear γH2AX S139 and pan-nuclear phospho-RPA32 S4/8 (Figs. 5c and S4B). After 24-h exposure to 500 nM AZD6738 alone, just 1.4% of WT MIA PaCa-2 cells were pan-nuclear for both markers, compared to 8.7% of ATM-KO cells (Fig. 5d). The combination of 500 nM AZD6738 and 10 nM gemcitabine induced pan-nuclear γH2AX S139 and phospho-RPA32 in both cell types but much more extensively in the ATM-KO cells (9.9% WT double positive vs 58.2% ATM-KO double positive after 24 h; Fig. 5e). Hoechst quantification confirmed that the majority of the cells with pan-nuclear damage had DNA contents between 2*n* and 4*n*, consistent with S-phase failure (Fig. S4C). Our observation of increased RC in the ATM-KO cells suggests that ATM protects against nuclear-wide fork collapse during ATRi/gem treatment.

### AZD6738 monotherapy causes growth delay in ATM-deficient PDAC xenografts, while combined treatment with gemcitabine induces regression

**Fig. 6:**
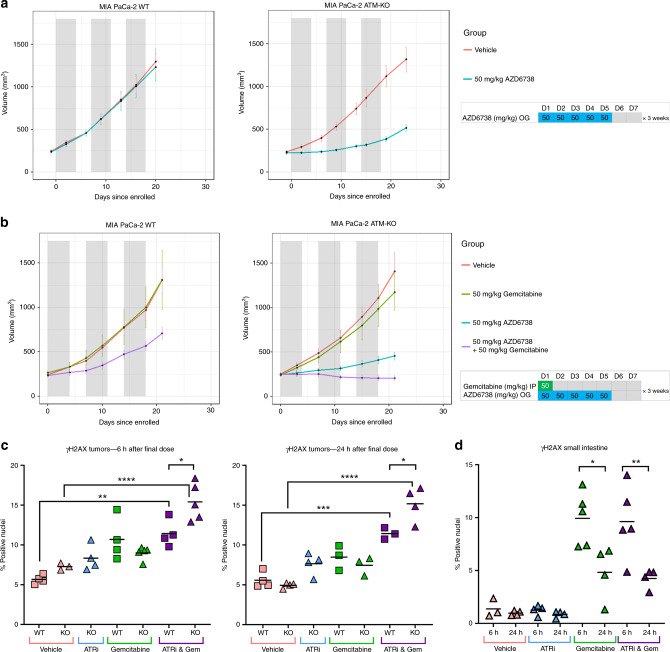
AZD6738 monotherapy causes growth delay in ATM-deficient PDAC xenografts, while combined treatment with gemcitabine induces regression. **a** Tumour volumes of MIA PaCa-2 WT and MIA PaCa-2 ATM-KO (clone B7) xenografts in NSG mice, with AZD6738 monotherapy at 50 mg/kg, OG, once daily, 5 days on and 2 days off. Grey bars denote the 5-day dosing cycles. Data, mean ± SEM. WT-Vehicle group, *n* = 10. WT-AZD6738 group, *n* = 9. ATM KO-Vehicle group, *n* = 9. ATM KO-AZD6738 group, *n* = 9. **b** AZD6738 and gemcitabine combination. AZD6738 was given at 50 mg/kg, OG, once daily, 5 days on and 2 days off. Gemcitabine was given at 50 mg/kg, IP, once per week on day 1 of each dosing cycle. Grey bars denote the 5-day dosing cycles. MIA PaCa-2 WT-Vehicle group, *n* = 8. WT-Gemcitabine group, *n* = 7. WT-AZD6738 and Gemcitabine group, *n* = 7. One mouse from the WT-Gemcitabine group and one from the WT-AZD6738 and Gemcitabine group dropped weight in the first week and their tumour volumes are not included in the mean values shown. MIA PaCa-2 ATM KO-Vehicle group, *n* = 8. ATM KO-Gemcitabine group, *n* = 9. ATM KO-AZD6738 group, *n* = 8. ATM KO-AZD6738 and Gemcitabine group, *n* = 9. **c**, **d** Nuclear γH2AX S139 positivity in the formalin-fixed tumour (**c**) and small intestine (**d**) samples from the combination study was assessed by IHC and quantified using the Halo software. Each point represents data from one histological section from an individual mouse. Horizontal bars denote the mean. A one-way ANOVA analysis was performed with Tukey’s multiple comparisons tests, *p ≤ 0.05, **p ≤ 0.01, ***p ≤ 0.001, ****p ≤ 0.0001.

Next, we assessed the efficacy of AZD6738 monotherapy in vivo. We treated NSG mice bearing either MIA PaCa-2 WT or MIA PaCa-2 ATM-KO subcutaneous xenografts, with 50 mg/kg AZD6738 (oral gavage, once daily, 5 consecutive days a week) for 3 weeks. No efficacy was seen in the ATM-proficient model, whereas in the ATM-KO tumours we observed a 61% TGI after 3 weeks of treatment (Fig. 6a). Subsequently, we assessed whether we could use low-dose gemcitabine as a sensitiser to AZD6738, to perhaps improve on the already impressive response seen with ATRi alone in the ATM-KO model. Regression is rare in MIA PaCa-2 xenograft studies, but adding gemcitabine (50 mg/kg, IP, once per week) to the 50 mg/kg AZD6738 schedule induced regression in the ATM-KO MIA PaCa-2 tumours (mean volume change after 3 weeks = −17.8%, 95% confidence interval −2.7% to −32.8%) and growth delay in the WT tumours (46% TGI) (Figs. 6b and S5A). As expected with this xenograft model, no metastases were identified in any group. The dose schedules used were well tolerated such that, across the two studies, just two mice experienced notable weight loss—one in the gemcitabine-alone group (1/17) and one in the ATRi/gem group (1/17) (Fig. S5B). Endpoint blood cell counts showed no significant difference between the ATRi/gem combination group and gemcitabine single agent (Fig. S5C).

IHC quantification of γH2AX percentage of positive nuclei in the tumours, fixed 6 h after the final dose, revealed an increase in the ATRi/gem groups compared to vehicle; mean γH2AX positivity increased from 5.7 to 11.4% in WT tumours with ATRi/gem (*p* < 0.01) and from 7.3 to 15.4% in ATM-KO tumours with ATRi/gem compared to vehicle (*p* < 0.0001) (Figs. 6d and S5D). γH2AX positivity was higher in the ATM-KO tumours following ATRi/gem treatment compared to the ATM-WT (*p* < 0.05). In both sets of tumours, the γH2AX positivity persisted for at least 24 h after dosing (Fig. 6d). Meanwhile, in the small intestine, the high γH2AX induced at 6 h by gemcitabine or ATRi/gem did not persist (Fig. 6e), indicative of DNA repair in normal tissue.

## Discussion

The application of precision medicine strategies, where therapies are assigned based on patient-specific tumour vulnerabilities, has the potential to improve outcomes for diseases such as PDAC. Having launched a phase I trial to assess the combination of AZD6738 and gemcitabine (ATRiUM, NCT03669601), we sought to investigate the potential utility of ATM loss as a predictive biomarker of response for ATRi/gem in PDAC. ATM and ATR have been reported to share a synthetic lethal relationship in some cancer types.^17–19,37,38^ However, it has become increasingly clear that most synthetic lethal interactions depend highly on the genetic background in which they are studied.^39^ By targeting ATM through multiple methods, our data indicate that complete loss of ATM function is necessary to sensitise PDAC cells to AZD6738 or the AZD6738 and gemcitabine combination. This has important implications, as it brings into question how best to assess ATM status in future clinical trials.

A recent example of a trial where ATM status was assessed is Study 39 (NCT01063517). This was a phase II efficacy study assessing the combination of olaparib and paclitaxel in gastric cancer patients, which identified a greater overall survival benefit in patients with “ATM-low” tumours.^40^ Here “ATM-low” tumours were defined as those with ≤10% ATM tumour cell nuclear staining, quantified by an IHC test using the ATM (Y170) antibody clone. In the follow-up phase III GOLD trial (NCT01924533), a new IHC reporter assay was developed that used the same antibody clone but with different assay configurations and reagents.^41^ The cut-off for “ATM-low” tumours was redefined as <25% of ATM tumour cell nuclear staining (to account for increased sensitivity of new assay); however, this trial failed to meet its primary endpoint and failed to confirm the survival benefit in “ATM-low” patients that had been observed previously.^42^ It remains unclear whether redefining the cut-off to a lower threshold would bring a different result. In relation to ATRi/gem in PDAC, our data would support the use of complete ATM loss (i.e. 0% ATM tumour cell nuclear staining), as opposed to <10 or 25% staining, since only ATM-null cells appeared to be hypersensitive. This leads to the question of how to identify ATM-null tumours in a clinical setting. Such tumour samples have been identified previously, including in the very first phase I dose escalation trial of an ATR inhibitor, which identified a colorectal cancer patient with 100% ATM loss by IHC, who showed a complete response (19+ months) to M660 monotherapy.^43,44^ Evaluating ATM functionality by IHC, using markers such as phospho-ATM and phospho-RAD50,^34^ in conjunction with total ATM could add further depth to ATM status assessments; however, this would likely require patients to be challenged with a DSB-inducing agent prior to biopsy to be most informative. The presence of germline or somatic ATM mutations, combined with a low IHC score, would add confidence that a patient’s tumour is indeed ATM negative, thus incorporating next-generation sequencing (NGS) in parallel with IHC could improve clinical biomarker assessments.^45^ On the whole, if precision medicine techniques are to be implemented successfully, it is becoming increasingly apparent that the field will have to be more precise and detailed in the characterisation of patients and their tumours, and multi-modal assessments of patient samples may facilitate this.

As well as the important distinction between ATM-low and ATM-null, our data also reveal further insight into the mechanism of how AZD6738 and gemcitabine synergise to induce cell death. The increase in nuclear RPA over time upon ATRi/gem exposure, plus the accumulation of S-phase cells pan-nuclear for DNA damage and replication stress markers, suggests that the combination induces RC (Fig. 5). During this catastrophic fork collapse, Chk1 and Chk2 are phosphorylated by DNA-PK, but the lack of clear ATRi/DNAPKi synergy and the inability for DNA-PKi to significantly potentiate ATRi/gem (Fig. 4g), suggests that these DNA-PK-driven signals may be bystander events during RC. The fact that ATM-deficient cells are hypersensitive to ATRi/gem-induced RC indicates that ATM plays a critical role in protecting against this widespread fork collapse. A recent study found that topotecan- or olaparib-induced breakage of replication forks is lethal in ATM-deficient models due to the induction of toxic non-homologous end joining (NHEJ).^23^ Their data suggested that the toxic NHEJ was mediated by XRCC4 and ligase IV, but not DNA-PK. Here we found that DNA-PKi did not significantly potentiate nor attenuate the effect of ATRi/gem in WT or ATM-KO cells. Going forward, it will be interesting to interrogate whether the hypersensitivity of ATM-null PDAC cells to ATRi/gem is also due to toxic NHEJ.

Minimising toxicity is a major challenge when combining DDRi and chemotherapy in the clinic. Most phase I trials of this type are designed such that the DNA-damaging cytotoxic is administered at its standard-of-care dose—i.e. at the expected maximum tolerated dose—and the DDRi is titrated at increasing levels. As a result, any increase in toxicity will immediately become dose limiting and the true potential of these combinations may not be fully explored. The ATRiUM trial design is unique, in that it is using a model-based approach to guide dose escalation, starting each drug at dosages that are <100% of the predicted or actual single-agent dose. In essence, the aim is to use the gemcitabine as a sensitiser to AZD6738, rather than simply administering the cytotoxic at its typical maximum dosage. In the preclinical study reported here, we demonstrated that this strategy can indeed be effective, particularly in an ATM-deficient setting. We first found that 50 mg/kg AZD6738 monotherapy, once daily for 5 consecutive days a week, induced significant growth delay in the ATM-null tumours. Rather than introduce gemcitabine at its typical preclinical single-agent dose of 100 mg/kg twice per week,^46^ we used a comparatively low gemcitabine dose of 50 mg/kg, once per week, to allow pharmacodynamically effective dose schedules of AZD6738. This regimen induced growth delay in the ATM-WT and tumour regression in the ATM-KO tumours (Fig. 6b), thus demonstrating proof of principle that low-dose gemcitabine can be used as a sensitiser to AZD6738 in vivo.

As well as assessing the safety, tolerability and preliminary antitumour activity of ATRi/gem in a novel model-based approach, the ATRiUM trial is incorporating IHC assessment of baseline ATM, NGS of patient DNA and on-treatment biopsies. This will enable some of the conclusions we have made in this study to be scrutinised clinically. This could steer the design of future precision medicine-based trials that will examine the promise that ATM shows as a predictive biomarker of response for AZD6738 and gemcitabine combination therapy in PDAC.

## Supplementary information

Supplementary Material

## Data Availability

All original data are archived and stored at the Cancer Research UK Cambridge Institute, Cambridge, UK. Cell lines generated in this study—MIA PaCa-2 ATM-KO (clones B4, B7, C4) and controls—will be made available to other researchers upon request.
