# Supplementary material for: Complete loss of ATM function augments replication catastrophe induced by ATR inhibition and gemcitabine in pancreatic cancer models
Source: Br J Cancer. 2020 Aug 3;123(9):1424–36. doi: 10.1038/s41416-020-1016-2 (PMC7591912; doi:10.1038/s41416-020-1016-2)
Supplement: Supplementary file 1 — Supplementary Material [file 41416_2020_1016_MOESM1_ESM.pdf]

SUPPLEMENTARY MATERIAL

Figure S1. Growth inhibition and synergy analysis of the ATRi and ATMi combination in panel of human and mouse cell lines

Cells were treated with increasing concentrations of AZD6738 (ATRi) and AZD0156 (ATMi) for 72 hours. Left = growth as % of solvent control (as assessed by SRB assay), mean ± SD, n=3. Right = Combenefit synergy score (Bliss and Loewe).

Figure S2. Characterisation of ATM-KO MIA PaCa-2 CRISPR clones

A. From genomic DNA, the region around the sgATM-Cas9 target site was amplified by PCR and sent for Sanger nucleotide sequencing. Chromatograms were deconvoluted using the Synthego ICE web tool (ice.synthego.com) which generates plots showing the relative prevalence of indels in each sample (i.e. in each clone). **B.** AZD6738 and gemcitabine GI_{50} values (the concentration that inhibits growth by 50% relative to control) for MIA PaCa-2 CRISPR clones. **C.** Gemcitabine dose-response curves of MIA PaCa-2 CRISPR clones. Assay duration was 72-hours. Each point represents the mean of three independent experiments ± SEM

Figure S3. Growth inhibition and synergy analysis of the ATRi and DNA-PKi combination in panel of human and mouse cell lines

A. Cells were treated with increasing concentrations of AZD6738 (ATRi) and AZD7648 (DNA-PKi) for 72 hours. Left = single agent dose response curves. Middle = growth as % of solvent control (as assessed by SRB assay), mean ± SD, n=3. Right = Combenefit synergy score (Bliss and Loewe). **B.** AZD76478 (DNA-PKi) dose response curves in WT and ATM-KO MIA PaCa-2 (clone B7). **C** Effect of DNA-PKi on ATRi sensitivity in WT and ATM-KO MIA PaCa-2 Assay duration was 72-hours. Each point represents the mean of three independent experiments ± SEM. **D.** Effect of DNA-PKi on ATRi/gem sensitivity in ATM-KO MIA PaCa-2. Assay duration was 72-hours. Each point represents the mean of three independent experiments ± SEM.

Figure S4. ATRi/gem-induced replication catastrophe in MIA PaCa-2 cells

A. DNA content of MIA PaCa-2 WT and MIA PaCa-2 ATM-KO cells following 24-hour exposure of the indicated drugs. Left = Representative DNA content histograms, x = FxCycle Violet fluorescence (DNA content), y = cell count. Right = cell cycle distribution. Each bar represents the mean of 3 independent experiments ± SD. **B.** Representative images showing the pan-nuclear emergence of yH2AX S139 and phospho-RPA32 S4/8 upon 24-hour exposure to 500 nM AZD6738 and 10 nM gemcitabine. The images shown were acquired using a 20x objective lens. **C.** Quantitative image-based cytometry was used to assess the DNA content (Hoechst 33342, x axis) of cells positive for yH2AX S139 (y axis) following ATRi/gem exposure. ATRi = 500 nM AZD6738. GEM = 10 nM gemcitabine. Images were acquired using the Operetta CLS High-Content Analysis System and analysis performed using Harmony 4.5 software. The resulting data were imported into FlowJo as CSV files to generate the pseudo-colour plots shown.

Figure S5. ATRi/gem *in vivo* xenograft assessments

A. Changes in individual tumour volume from start to the end of the combination study (day 0 to day 21), as a percentage of starting volume. A one-way ANOVA analysis was performed (with multiple comparisons tests assuming unequal variances), * indicates $p \leq 0.05$, **$p \leq 0.01$, ***$p \leq 0.001$, ****$p \leq 0.0001$. **B.** Individual body weights during treatment, as a percentage of the starting weight upon enrolment, for NSG mice bearing MIA PaCa-2 WT or ATM-KO (clone B7) xenografts. Both sets of mice from Figure 6A (monotherapy study) and Figure 6B (subsequent combination study) are plotted. Just two mice showed significant weight loss, Mouse#220_42 (Gemcitabine group) and Mouse#220_42 (ATRi/gem group). **C.** Blood was taken at endpoint and run on the Mythic 18 Haematology Analyser. The title of each sub-plot denotes the blood component measurement and its units. RBC = red blood cells, PLT = platelets, HGB= haemoglobin; WBC = white blood cells, Lym = lymphocytes, Mon = monocytes, Gra = granulocytes. Each point represents the reading from one mouse. Bars = mean ± SD. **D.** Representative IHC images of γH2AX staining on formalin-fixed tumour samples from the ATRi/gem combination study (quantification in Figure 6C). Samples shown were fixed 6 hours after the final dose. Images were viewed at 5X magnification in Halo software and compiled into the grids displayed using the Figure Maker tool within Halo.

Supplementary Figure S1

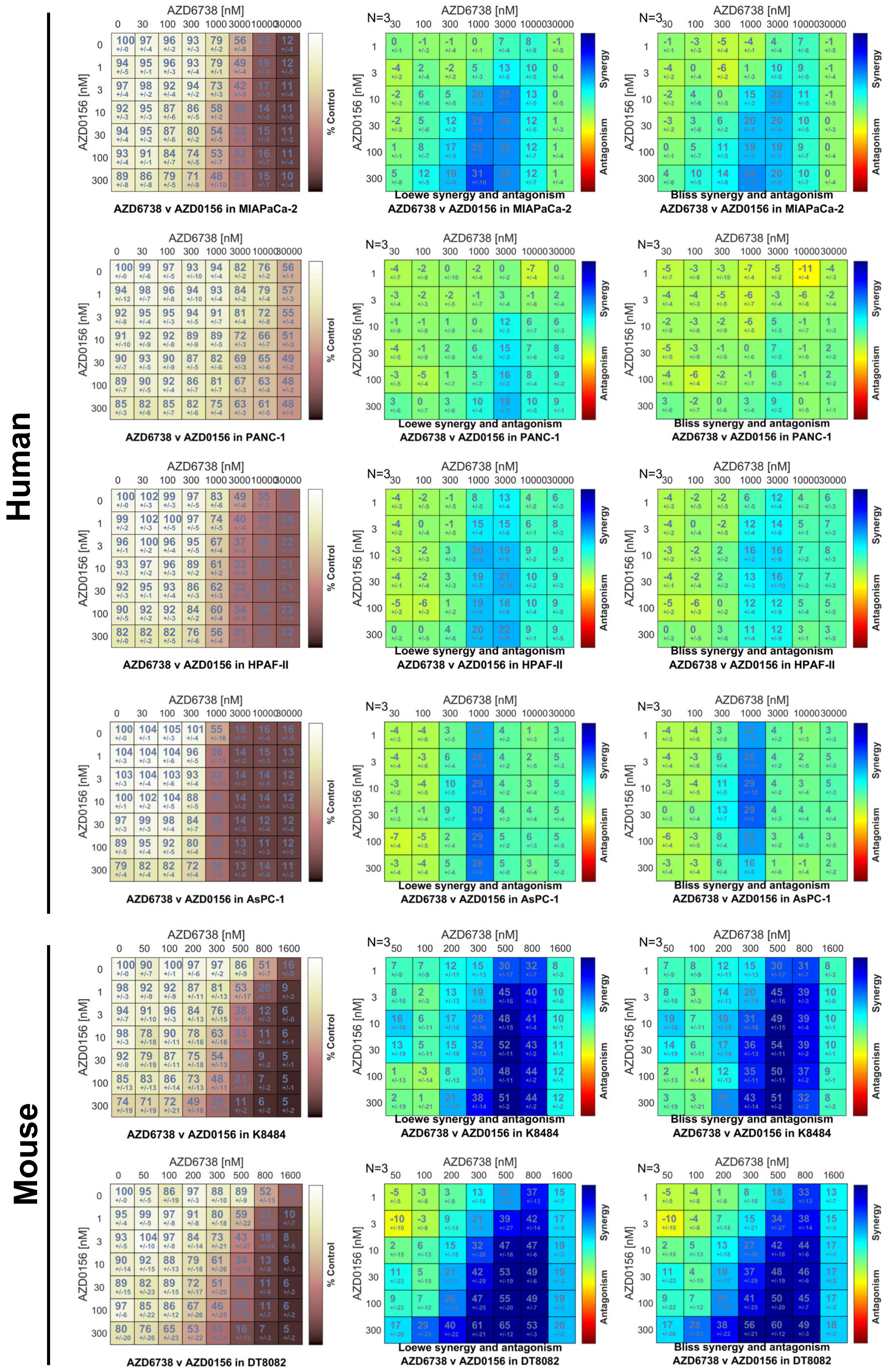

Supplementary Figure S2

A

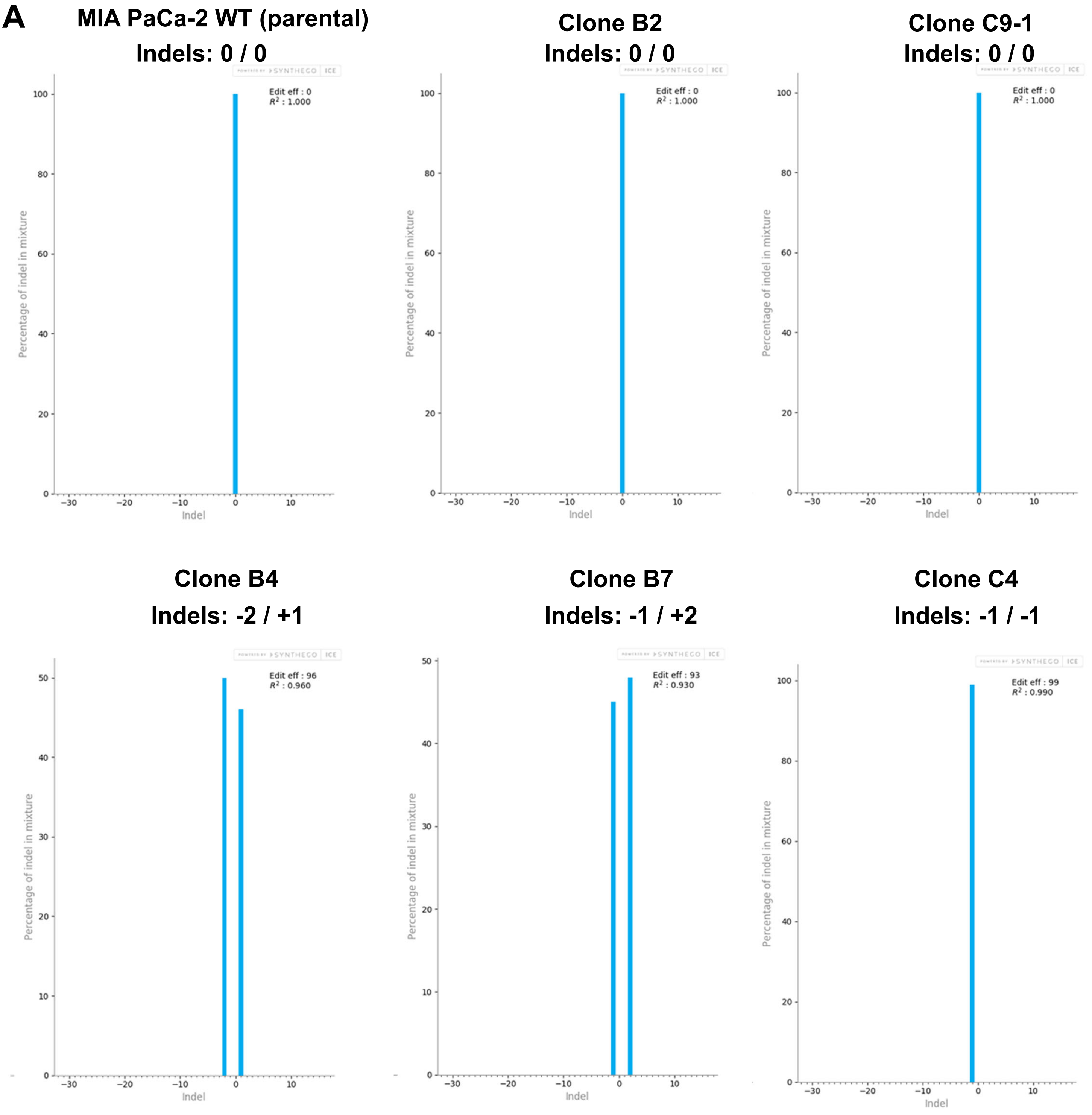

B

Clone name	ATM status	AZD6738 GI50 (nM)	95% confidence interval (nM)	Mean GI50 (nM)
WT (parental)	positive	2819	2486 to 3227	2728
B2	positive	2909	2412 to 3582	
C9_1	positive	2458	2106 to 2911	
B4	null	405	362 to 477	372
B7	null	373	325 to 430	
C4	null	337	287 to 398	

Clone name	ATM status	GEM GI50 (nM)	95% confidence interval (nM)	Mean GI50 (nM)
WT (parental)	positive	22	15 to 31	26
B2	positive	30	25 to 37	
C9_1	positive	27	22 to 34	
B4	null	7	5 to 9	10
B7	null	11	9 to 14	
C4	null	11	10 to 13	

C

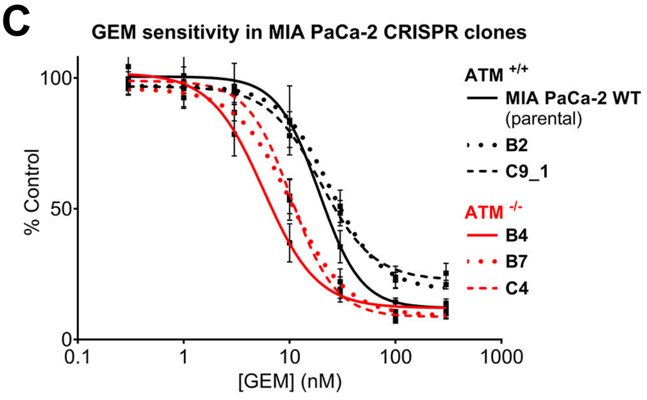

Supplementary Figure S3

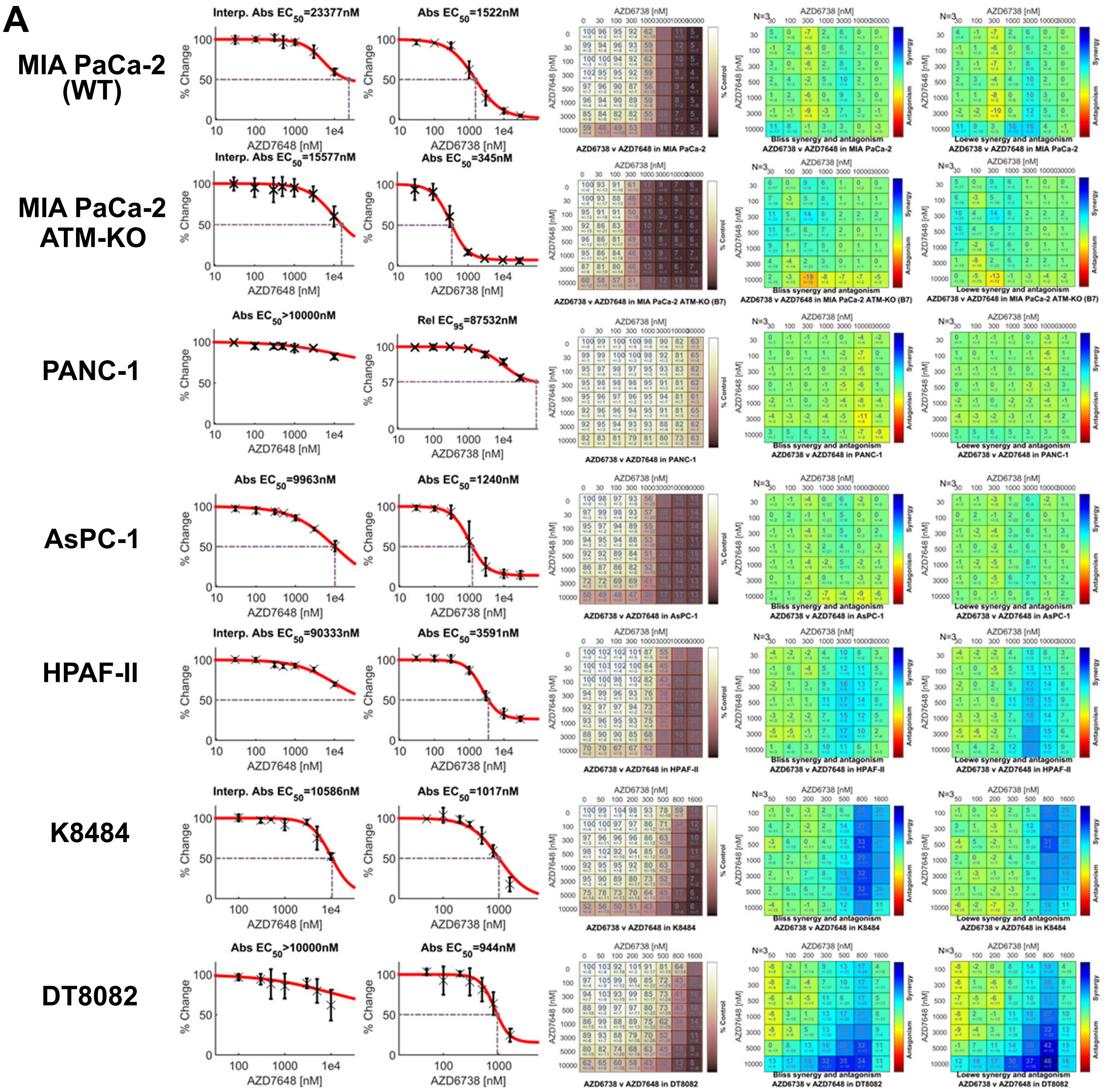

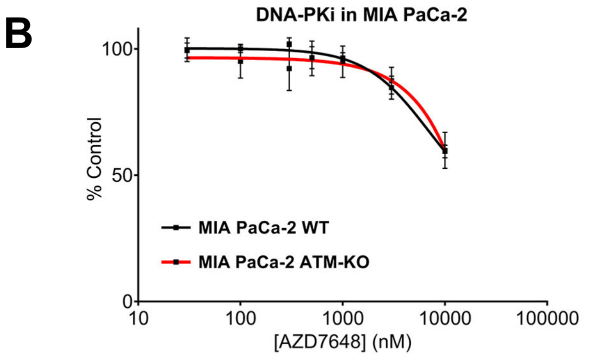

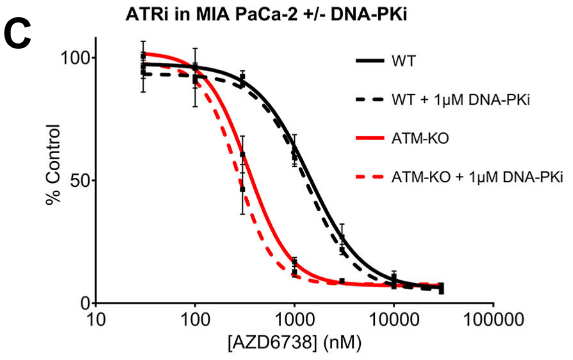

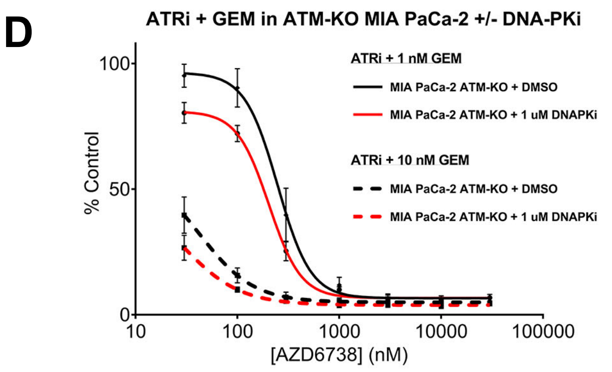

Supplementary Figure S4

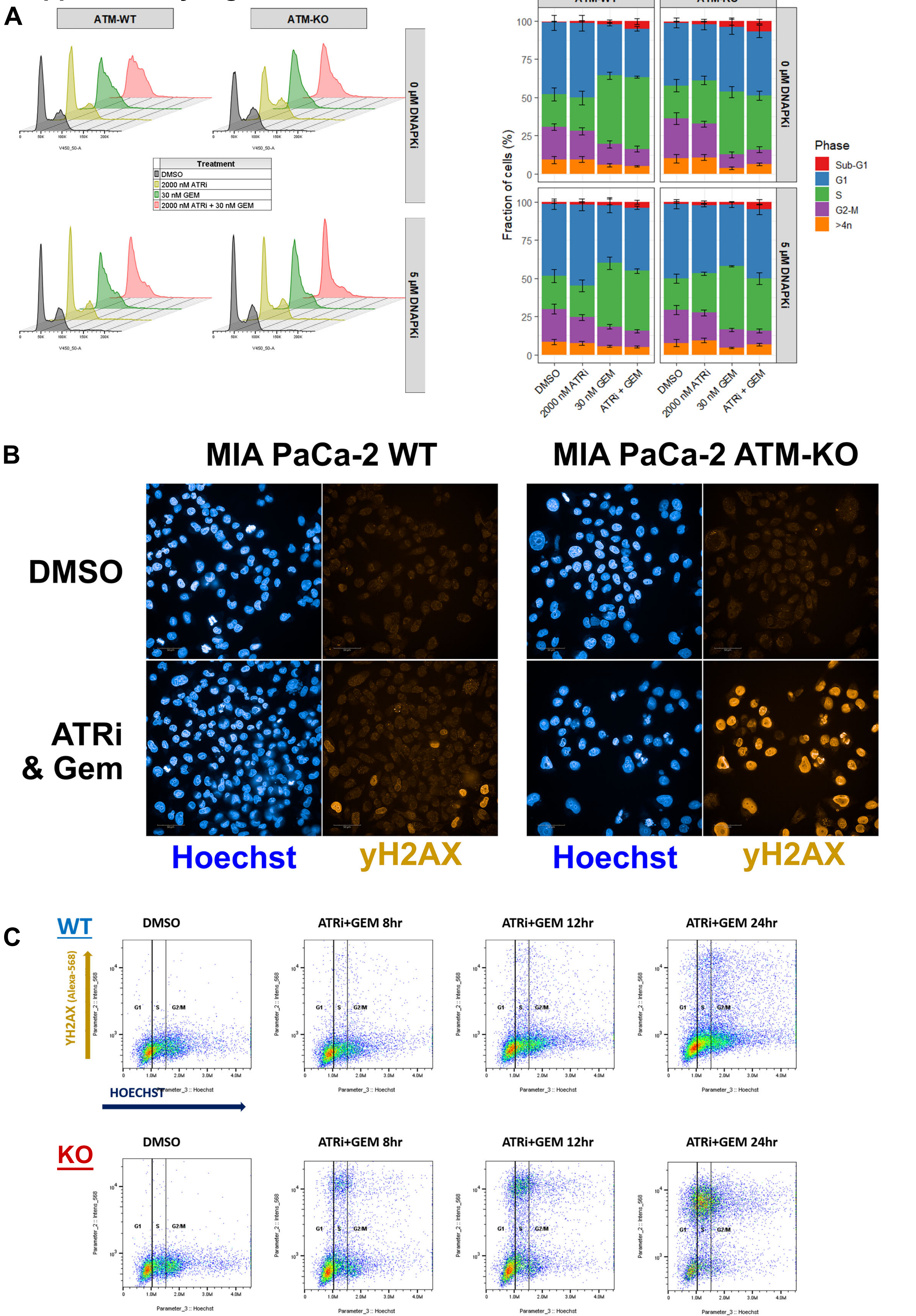

Supplementary Figure S5

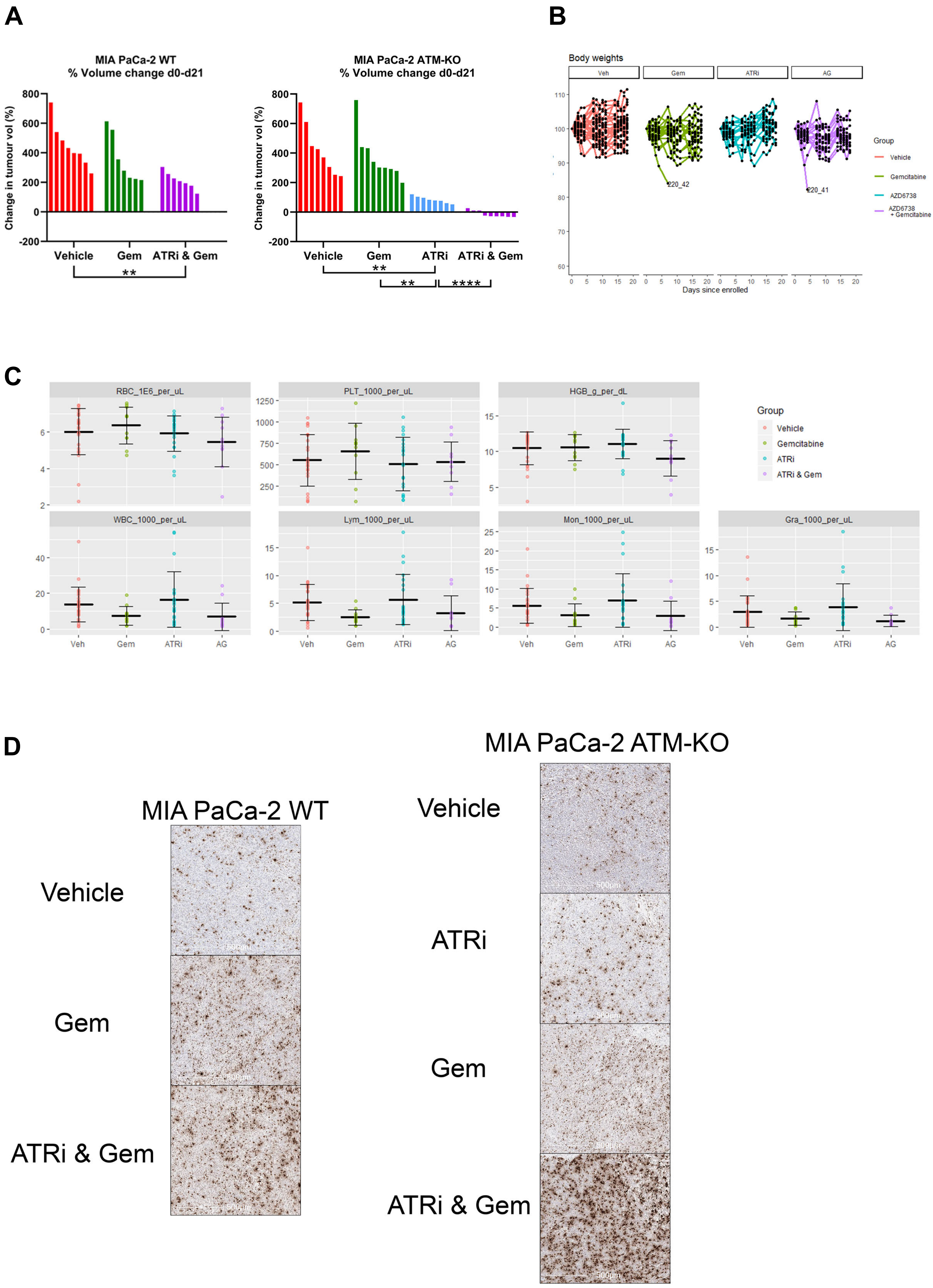

Spiral randomisation method for tumour bearing mice

1. Measure tumour volumes as close as possible to the day the first dose is required.

2. Discard any outliers (with unusually large tumour or those whose tumour is smaller than the initiation criteria, with double tumours, etc)

3. In excel, sort the mice by tumour volume, largest to smallest

4. Run down the sorted list from largest tumour, assigning groups in a spiral. e.g. for 3 groups, assign to 1,2,3,1,2,3, etc

5. Re-sort based on assigned group, and check for balance by calculating mean tumour volume in each group

6. Assign Treatments to Group numbers, ideally blinded as in B.2, above. Do not assign Group 1 to the control (vehicle) arm because that group will always have the largest tumour.

Animal Experiments Meta Data

Mouse details		
	generic Mouse strain/line name	NSG
	Supplier	Charles River
	gender	female
	age at start of Study procedure	purchased at 42-55 days
	Full mouse strain/substrain name	NOD.Cg-*Prkdc*^{scid} *Il2rg*^{tm1Wjl}/SzJ
Housing and Husbandry		
	Building/room housed	B51
	Room temperature	Set at 21 deg C +/- 2 deg C; humidity 55% +/- 10%
	light/dark cycle	12 hour light 7am to 7pm
	Type of cage	Individually ventilated cages
	Bedding	Aspen wood chip, with substrate either "nestlets"
	Food (detail of diet)	Standard is LabDiet 5R58, irradiated
	Water	bottle, ad libitem
	Environmental enrichment	fun tunnels and chew blocks
	Special conditions	None
